# Exploring Mass Transfer in Mesoporous Zeolites by NMR Diffusometry

**DOI:** 10.3390/ma5040699

**Published:** 2012-04-20

**Authors:** Dirk Mehlhorn, Rustem Valiullin, Jörg Kärger, Kanghee Cho, Ryong Ryoo

**Affiliations:** 1 Faculty of Physics and Earth Science, University of Leipzig, Linnèstr. 5, Leipzig D-04103, Germany; E-Mails: mehlhorn@physik.uni-leipzig.de (D.M.); kaerger@physik.uni-leipzig.de (J.K.); 2 Department of Chemistry and Graduate School of Nanoscience and Technology (WCU), KAIST, Daejeon 305-701, Korea; E-Mail: chokanghee@kaist.ac.kr

**Keywords:** mesoporous zeolites, diffusion, surface barriers, NaCaA, propane, NMR

## Abstract

With the advent of mesoporous zeolites, the exploration of their transport properties has become a task of primary importance for the auspicious application of such materials in separation technology and heterogeneous catalysis. After reviewing the potential of the pulsed field gradient method of NMR (PFG NMR) for this purpose in general, in a case study using a specially prepared mesoporous zeolite NaCaA as a host system and propane as a guest molecule, examples of the attainable information are provided.

## 1. Introduction

In modern technologies, nanoporous materials find widespread application in shape-selective catalysis and in mass separation by molecular sieving and selective adsorption. All these processes are prompted by the intimate contact between the guest molecules and the host surface [1,2,3], which is realized in the best possible way if the pore sizes are close to the molecular dimensions. This closeness, however, may in turn lead to a dramatic reduction in the transport rate of the guest molecules. The gain in value-added products can never exceed the rate of their escape out of the nanoporous particles where—owing to separation and/or conversion—they originate from. The reduction in transport rates is often accompanied, therefore, by a distressing reduction in process performance [4,5,6]. 

A most promising way to circumvent the problems emerging from the contradicting requirements for high guest mobility and intimate host-guest interaction has opened up with the advent of hierarchical porous materials [7,8,9,10,11]. In addition to their content of micropores for maintaining their functionality for mass separation and conversion, these materials are traversed by a network of mesopores which accelerate mass transfer between the micropores as the “active” parts of the host particles and their surroundings. 

For evaluating mass transfer in complex systems, the pulsed field gradient technique of NMR (PFG NMR, also referred to as the PGSE (pulsed gradient spin echo) NMR) has proved to be a most versatile and sensitive tool [12,13,14,15]. As an *in-situ* technique, it allows to record the redistribution rate of the molecules within the sample over distances of typically micrometers and time spans of typically milliseconds till seconds, without any interference with the intrinsic processes of molecular propagation. 

With its application to zeolites [16], for quite a number of zeolitic host-guest systems PFG NMR has given rise to a totally new view on the guest mobility in zeolite micropores [6,17]: The diffusivities determined with the novel options provided by PFG NMR were found to exceed previously measured and generally accepted ones in some cases by even as much as several orders of magnitude. Today, in many cases these differences may be attributed to the existence of additional transport resistances like surface barriers [18] and/or transport resistances in the zeolite bulk phase [19,20] acting in addition to the diffusion resistance of the genuine micropore space [21]. 

The techniques of molecular modeling, which have vividly developed in that time, nicely reproduced the diffusivity data determined by NMR [22,23,24]—in complete accordance with the underlying assumption that mass transfer occurs under the sole influence of the genuine pore space. In this respect, quasi-elastic neutron scattering (QENS) offers even better prospects for comparison with the modeling data [25]. By considering mass transfer phenomena over nanometers [26] (rather than micrometers, as PFG NMR) QENS remains essentially completely unaffected by any of the above mentioned resistances acting in addition to the genuine diffusion resistance of the pore space.

Depending on the chosen pulse sequence of measurement (see Section 2.1) and the system under study, PFG NMR is able to vary the distance over which the diffusion path is followed, covering a range from below 100 nm up to 100 μm. It is due to this possibility that, by recording over sufficiently short diffusion path lengths, PFG NMR is able to sense molecular diffusion in the genuine zeolite pore space, yielding complete agreement with QENS [27,28] or the modeling results [29,30]. With increasing diffusion path lengths, however, PFG NMR becomes more and more sensitive to further mechanisms of mass transfer control. It is this ability which makes PFG NMR particularly valuable for an in-depth assessment of mass transfer phenomena in hierarchical materials. 

An important pre-condition of the first successful PFG NMR measurement of intracrystalline diffusion in zeolites was the availability of sufficiently large zeolite crystallites. Only owing to this material it was possible to demonstrate [31,32] that early PFG NMR measurements of water in commercial FAU-type zeolites [33] did yield an effective (the “long-range”) rather than the true intracrystalline diffusivity. This novel type of measurement has become possible thanks to the unique zeolite material [34], synthesized by Professor Sergey Petrovich Zhdanov in Leningrad, in the Soviet Union, which was available in Leipzig, on the same side of the iron curtain at that time, and which has given rise to a number of papers dealing with intracrystalline diffusion in zeolites [35,36] as well as with diffusion under the special conditions of chemical reactions [37]. We dedicate this paper to the memory of Professor Zhdanov on the occasion of the 100th anniversary of his birthday on 18 April 2012, recognizing the most productive collaboration between his group and the group in Leipzig, in deep appreciation of his contribution to the development in the field and of the eventual break-down of the iron curtain. 

## 2. Experimental Section

### 2.1. PFG NMR Diffusion Measurements

Diffusion measurement by nuclear magnetic resonance (NMR) is based on the fact that the nuclei of most atoms, in particular protons, possess a magnetic moment. By adopting the quasi-classical interpretation of nuclear magnetism, the magnetic moment may be understood as a consequence of the rotation of the nucleus (the nuclear “spin”), giving rise to a magnetic moment as observable with any circular current. This combination of a magnetic and mechanic moment typical of nuclear spins makes them, under the influence of a magnetic field, behave like a gyroscope under the influence of gravity: Instead of equilibrating into the direction of the magnetic field they perform a precessional motion about the direction of the magnetic field. The frequency of this rotation (the “Larmor frequency”) may be shown to be proportional to the intensity *B* of the magnetic field [38,39]:

*ω* = *γB* (1)

The quantity *γ*, referred to as the gyromagnetic (or magnetogyric) ratio, is a characteristic quantity of the given nucleus (for protons ≈ 2.675 × 10^8^ T^−1^s^−1^) and represents, in the quasi-classical model, nothing else than the ratio between the magnetic and mechanic momenta of the nucleus.

The signal observed in NMR is brought about by the magnetization in the plane perpendicular to the externally applied magnetic field, rotating (“precessing”) with the Larmor frequency as given by Equation (1), and is nothing else than the voltage induced by the rotating magnetization in a receiver coil, appropriately circumscribing the NMR samples, whose spins are under consideration. 

**Figure 1:**
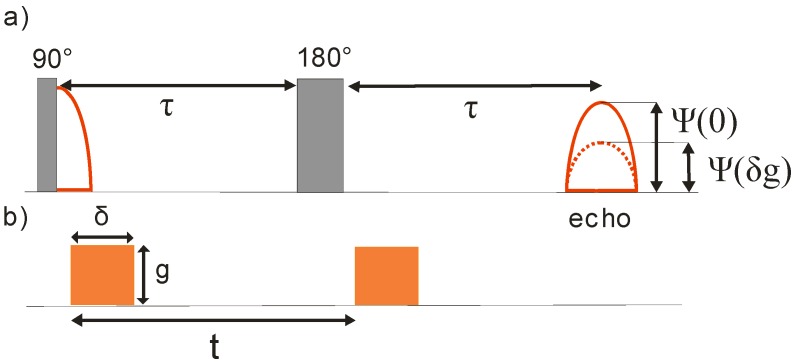
Pulse sequence for generating the NMR Hahn echo with (**a**) the two observable main signals (the free induction decay following the π pulse, and the Hahn (spin) echo) and (**b**) the field gradient pulses, applied in the basic PFG NMR experiment for self-diffusion measurement.

Under equilibrium, the magnetization is parallel to the direction of the magnetic field. Figure 1a displays a simple scheme designed for the formation of nuclear magnetism in the plane perpendicular to the magnetic field, the Hahn spin-echo sequence [39,40]. It consists of two radio-frequent (rf) pulses (operating with the Larmor frequency), with durations adjusted in such a way that, around a direction perpendicular to the constant magnetic field, the magnetic moments of each individual spin (more strictly: their expectation values) are turned by 90° by the first one (the π/2 pulse) and by 180° by the second one (the π pulse), which is applied after a certain time interval *τ*.

If applied under equilibrium conditions, the first rf pulse turns each individual spin and, hence, their total sum, the magnetization, from its equilibrium value, *i.e.*, from an orientation parallel to the magnetic field, into the plane perpendicular to this direction. Just as each individual spin, also this magnetization rotates (precesses) about the direction of the magnetic field, with the Larmor frequency (Equation (1)). In the receiver coil, this rotating magnetization induces an rf voltage of frequency *ω* which is displayed in the figure and referred to as the free induction decay. The fast decay of this signal is caused by the omnipresent, unavoidable differences in the intensity of the magnetic field over the sample. These (even rather small) differences in the magnitude of *B* lead, via Equation (1), to differences in the precessional frequencies and, hence, to an increasing spread in the orientations of the individual spins with a corresponding decrease of their vector sum, the magnetization, leading eventually, to its extinction. 

By the application of a second, appropriately chosen rf pulse (a “π pulse”) Erwin Hahn has designed an easy means to recreate the vanished signal [40]. As a consequence of their rotation by 180° with respect to an (arbitrary) direction perpendicular to the direction of the constant magnetic field, now those spins which are situated at positions with the lowest magnetic fields and which, therefore, are oriented “behind” the mean value of the orientation of the individual spins, are now brought in front of this average. *Vice versa*, the “fastest” spins are now “behind” the average. After application of the π pulse, it takes therefore exactly once again the time *τ* which has elapsed between the π/2 and the π pulse, before all spins point to the same direction giving, once again, rise to a signal, the “spin (or Hahn) echo”. 

It is illustrated in Figure 1a that the magnitude of the echo maximum is smaller than the intensity of the free induction decay. This is a consequence of the so-called transverse nuclear magnetization (whose time constant is referred to as *T*_2_), representing the indispensable process of equilibrium approach (where the magnetization component perpendicular to a constant magnetic field has to be zero).

In the pulsed field gradient method (PFG NMR), the most powerful technique of diffusion measurement by NMR, by the application of the so-called field gradient pulses, over two short time intervals of duration *δ* (Figure 1b) the magnetic field acting on the sample is made extremely inhomogeneous. Thus, during the gradient pulses, the magnetic field at position *z* becomes

*B* = *B*_0_ + *gz* (2)

where the magnetic field gradient (pointing, in general, in the direction of the axis of the sample tube) is chosen to determine the *z* direction. 

Like the natural field inhomogeneities (whose influence is eliminated in the spin echo experiment), these field gradient pulses lead to differences in the Larmor frequencies, depending on the given location *z*. If the molecules (and hence the spins under consideration) may be assumed to be fixed in space, also their influence is clearly eliminated in the spin echo experiments. The situation becomes, however, totally different if, as a consequence of their diffusional motion, the molecules are situated at different positions during the application of the two gradient pulses. In this case, at the time of the spin echo each individual spin does contribute to the vector sum, *i.e.*, to total magnetization and, hence, to the observable spin echo, with only the cosine of the angle between its actual orientation and the mean orientation. By combining Equations (1) and (2) this difference is easily found to be equal to *γδgz* where *z* denotes the distance over which the molecule under consideration have been shifted during the time *t* between the two field gradient pulses—where, for simplicity, the duration *δ* of the field gradient pulses is assumed to be negligibly small in comparison with the “observation” time *t*, *i.e.*, the time interval between the two gradient pulses. Under the influence of the applied field gradient pulses, molecular redistribution within the sample is immediately seen to give rise to an attenuation of the NMR spin echo (broken line in Figure 1a).

(3) $$M(\delta g)/M(0)\equiv \psi \left(\gamma \delta g,t\right)=\int_{-\infty }^{\infty }P(z,t)\cos\left(\gamma \delta gz\right)dz$$

*P*(z,*t*) denotes the probability that, during time *t*, an arbitrarily selected molecule within the sample is shifted over a distance *z*. It is referred to as the mean propagator and represents the key function for correlating the PFG NMR experiment with the intrinsic diffusion phenomena [41]. This interrelation between the signal attenuation curve *ψ*(*γδgz*) and the mean propagator gives rise to the matchless versatility of PFG NMR for the exploration of molecular mass transfer in complex systems, notably in porous materials [42]. 

The mechanism leading to signal attenuation does, in principle, also apply for the omnipresent inhomogeneities of the magnetic field referred to on describing the Hahn spin echo experiments. Since the externally applied field gradients exceed the internal ones by several orders of magnitude, however, this influence may, generally, left out of consideration.

It is important to note that the interaction energy between the nuclear spins and the externally applied magnetic field is negligibly small in comparison with the thermal energy (*kT*). With the exception of measurements at extremely low temperatures, NMR experiments may therefore be considered to proceed without any disturbing influence on the intrinsic processes going on in the sample under study, allowing true “*in-situ*” measurements. 

For molecules in an infinitely extended, isotropic medium the propagator may be shown to be represented by a Gaussian [6,15,43]

(4) $$P(z,t)=\frac{1}{\sqrt{4\pi Dt}}\exp\left\{-\frac{z^{2}}{4Dt}\right\}$$

with the mean square width (the molecular mean square displacement) increasing linearly with time following the Einstein relation

(5) $$\langle z^{2}(t)\rangle \equiv \int_{-\infty }^{\infty }z^{2}P(z,t)dz=2Dt$$

The quantity *D* appearing in Equations (4) and (5) is the coefficient of self-diffusion (or the self-diffusivity, in general shortly referred to). The validity of Equation (5) is the pre-condition for the occurrence of “normal” diffusion [44,45]. Completely equivalently, the occurrence of normal diffusion may as well be based on the validity of Fick’s 1st law, implying proportionality between the gradient of labelled molecules and their flux density. The factor of proportionality appearing in this relation may be shown to be nothing else than the self-diffusivity appearing in Equations (4) and (5) [6,43].

For appreciating the information provided by PFG NMR it is important to rationalize that, at sufficiently small concentrations (*i.e.*, in the limiting case where the mutual interaction of the diffusing molecules becomes negligibly small), the self-diffusivity coincides with the coefficient of transport diffusion, *i.e.*, the factor of proportionality between a macroscopic concentration gradient and the net flux to which it has given rise. It is this latter process which is relevant under the conditions of molecular uptake and release so that the information provided by PFG NMR is of immediate importance for the microdynamic phenomena during the technological application of these materials (occurring under non-equilibrium conditions, *i.e.*, with concentration gradients, rather than under equilibrium).

By inserting Equation (4) into Equation (3), the attenuation of the spin echo due to diffusion under the presence of magnetic field gradient pulses is found to be

(6) $$\psi \left(\gamma \delta g,t\right)=\exp\{-\gamma^{2}\delta^{2}g^{2}Dt\}=\exp\left\{-\frac{1}{2}\gamma^{2}\delta^{2}g^{2}\langle z{\left(t\right)}^{2}\rangle \right\}$$

with the second equation resulting by the use of Equation (5). It is important to note that this second relation does also provide a useful approach of the spin-echo attenuation under conditions deviating from those relevant for normal diffusion. 

An important example for such a deviation is given by the conditions of restricted diffusion, namely in cases where molecular propagation is confined to a certain range, e.g., the interior of the individual crystallites of a sample. Also under such conditions it is useful to use Equation (6) in combination with Equation (5) which now defines an “effective” diffusivity (which, clearly, coincides with the true diffusivities as soon as the measuring conditions for normal diffusion are fulfilled). 

We shall make use of such effective diffusivities in the limiting case of sufficiently large observation times for molecular displacements confined to a sphere of radius *R* (considered as an approximation for the shape of an individual zeolite crystal). The effective diffusivity under such conditions is given by the relation [6]:

(7) $$D_{\text{restr.}}=\frac{R^{2}}{5t}$$

If the nanoporous material under study consists of small particles (the zeolite crystallites) of finite size, the PFG NMR attenuation curve has to be approached by a sum of two exponentials rather than by a single one:

(8) $$\psi \left(\delta g,t\right)=p(t)\exp\left\{-\frac{1}{2}{\left(\gamma \delta g\right)}^{2}{\langle z{\left(t\right)}^{2}\rangle }_{\text{intra}}\right\}+[1-p(t)]\times \exp\left\{-\frac{1}{2}{\left(\gamma \delta g\right)}^{2}{\langle z{\left(t\right)}^{2}\rangle }_{\text{long}-\text{range}}\right\}$$

here, the first term stands for diffusion of molecules inside the zeolite (intracrystalline diffusion) and the second term describes diffusion which occurs in the external space (intercrystalline diffusion).The function *p*(*t*) denotes the fraction of molecules which, during the observation time *t*, have remained within one and the same crystal.

In the first term of Equation (8) we easily recognize Equation (6). In the limit of sufficiently short observation times *t* and/or sufficiently large crystal radii, the amount of molecules leaving the crystals will be negligibly small (*i.e.*, *p*(*t*) ≈ 1) and the intracrystalline mean square displacement <*z*^2^(*t*)>_intra _shall be given by the Einstein relation, Equation (5), with *D* denoting the genuine intracrystalline diffusivities. As soon as, with increasing time and for sufficiently small crystals, an appreciable number of molecules is able to leave, during *t*, their crystallite, *p*(*t*) will decrease and, simultaneously, the mean square displacements of the molecules in the intracrystalline space shall be more and more affected by the finite size of the crystals. In this case, application of Equation (5) leads to an effective (intracrystaline) diffusivity rather than to the genuine diffusivity in the intracrystalline pore space. The upper limit of the effective diffusivity for mass transfer within the crystals is given by Equation (7). 

Simultaneously, an increasing amount of molecules is able to leave the crystals, giving rise to the second term in Equation (8). Corresponding with the notably larger diffusion path lengths in the intercrystalline space, the exponent of the second term notably exceeds that of the first one. 

In the common representation of the PFG NMR spin-echo attenuation as a logarithmic plot of the signal intensity versus the squared gradient pulse intensity (*δ*^2^*g*^2^) the two contributions to Equation (8) can be easily identified. This allows, in particular, the determination of the mean square molecular displacements <*z*^2^(*t*)>_intra_ within the crystals (from the slope of the second, slowly decaying component) and of the relative amount *p*(*t*) of molecules which have remained within the crystals (relative contribution of this second decay, easily to be determined by extrapolation to the ordinate).

The incorporation of a network of mesopores in hierarchical zeolites leads to a fundamental change in the mechanism of intracrystalline diffusion. In the ideal case of a purely microporous zeolite without intracrystalline transport resistances acting in addition to the micropore space, the intracrystalline diffusivity as observable by PFG NMR is clearly given by the micropore diffusivity *D*_micro_. It is this quantity which is also observed by QENS and which is expected to result from molecular modeling by molecular dynamics simulations within the force field exerted by the genuine pore space on the guest molecules. 

This situation is notably changed by the presence of a network of mesopores traversing the micropore space. Let us assume, for simplicity, that the guest molecules exchange sufficiently fast between the micro- and mesopore spaces so that the mean life time within one of these spaces is much smaller than the mean uptake or release time. In this case, by applying the fast-exchange model of PFG NMR [13,46,47,48], the intracrystalline diffusivity as accessible by PFG NMR is given by the relation

*D*_intra_ = *p*_micro_*D*_micro_ + *p*_meso_*D*_meso_ (9)

with *p*_micro(meso)_ and *D*_micro(meso)_ denoting, respectively, the relative number of molecules in the micro(meso)pores and their diffusivity. With Equation (9), the intracrystalline diffusivity is thus easily seen to be notably enhanced in comparison with the micropore diffusivity *D*_micro_ if (and only if!) *D*_micro_ is notably exceeded by the term *p*_meso_*D*_meso_, *i.e.*, by the product of the relative number of guest molecules in the mesopores and their diffusivity.

It is noteworthy that the very first PFG NMR diffusion studies with mesoporous zeolites were performed with water in the sodium form of those LTA-type zeolites which are also in the focus of the present study [49]. The most favorable water adsorption in such systems leads to rather small values of *p*_meso_ so that, under the conditions chosen in these studies, the second term in Equation (9) did not notably exceed the order of magnitude of the first one so that the effect of the mesopores on the rate of intracrystalline mass transfer remained quite modest [49]. In the present study this limitation is overcome by considering the diffusion of propane in the Ca-exchanged specimens of LTA, giving rise to much larger values of *p*_meso_ with, simultaneously, maintenance of the high values of *D*_meso_ characteristic for Knudsen flow through the mesopores [50,51,52].

Data analysis of PFG NMR diffusion measurements with nanoporous particles following Equation (8) has been referred to as the NMR tracer desorption (or tracer exchange) technique [13,53]. In fact, by determining the time dependence *p*(*t*) of the fraction of molecules which have not yet exchanged with the surroundings of the particle in which they were situated at time *t* = 0, one attains the same information as accessible by classical tracer exchange experiments [6,54], with the important differences that now the “observation” times are in the range of typically milliseconds rather than minutes and that any influence of extracrystalline mass transfer is definitely excluded. 

As a key quantity for analyzing the NMR tracer desorption curve *p*(*t*) it is useful to adopt the formalism of the statistical moments which, after having originally been introduced for analyzing the chromatographic response in packed columns [55,56], has most successfully been applied for the analysis of transient uptake and release as well as of tracer exchange curves [54,57,58]. In this formalism, the mean life time *τ*_intra_ of a guest molecule in the interior of a nanoporous particle is represented as the first statistical moment of the tracer exchange curve which is defined by the relation

(10) $$\tau_{\text{intra}}=\int_{0}^{\infty }p(t)dt$$

If molecular exchange is controlled by exclusively intracrystalline diffusion (*i.e.*, under the absence of any further transport resistances), the intracrystalline mean life time may be shown to be [6,54]

(11) $$\tau_{\text{intra}}^{\text{diff}}=\frac{R^{2}}{15D_{\text{intra}}}$$

For an order-of-magnitude estimate of the intracrystalline mean life times, we are going to approach the time dependence of the tracer exchange curve *p*(*t*) by an exponential

(12) $$p(t)\approx \exp(-\frac{t}{\tau_{\text{(intra)}}})$$

where, by inserting this relation into Equation (10), the time constant *τ*_(intra)_ is immediately seen to coincide with the intracrystalline mean life time. This particular form of the tracer exchange curve is completely correct only in the rather exceptional case that tracer exchange is controlled by surface barriers where, in addition the sizes and surface resistances (or, slightly less stringently and more correctly, the ratios between the radii and the surface permeabilities [6]) of all crystals are identical. However, given the relative small number of different values *p*(*t*_i_) (with *t*_i_ denoting the particular observation time) typically attainable in a PFG NMR experiment and the substantial uncertainty by which they are attained, this type of approach turns out to be, in general, totally sufficient for an order-of-magnitude estimate of the intracrystalline mean life time of the guest molecules within the sample. 

The precision of the analysis most significantly depends on the width of the interval over which the observation time *t* of the PFG NMR experiment can be varied. With Equation (6) it is easily seen that the magnitude of the parameters *δ* and *g* and the diffusivity *D* pose a lower limit on the observation time so that the exponent attains sufficiently large values (typically of order 1) for giving rise to a sufficiently pronounced signal attenuation. In our experiments, this lower time limit was of the order of 10 ms. Towards large values, the observation time for intracrystalline mass transfer is limited by the intracrystalline mean life time: Reliable measurements of intracrystalline transport phenomena are only possible if a sufficiently large fraction of the molecules under study remains, during the observation time, within one and the same crystal.

Another limitation is imposed on the observation times due to nuclear magnetic relaxation. In Figure 1, transverse nuclear magnetic relaxation was seen to lead to an attenuation of the NMR spin echo in comparison with its maximum possible value, given by the initial value of the free induction decay. The accuracy, with which the PFG NMR attenuation curve of the spin echo can be measured, clearly depends on the intensity *M*(0) of the signal without field gradients applied. The upper limit of the observation time in PFG NMR diffusion measurements by the Hahn echo is, therefore, closely related to the transverse nuclear magnetic relaxation time *T*_2_. 

This limitation can be overcome with the so-called stimulated echo [6,47,59]. Instead of a single π pulse, the stimulated echo is generated by applying a pair of π/2 pulses between the two field gradient pulses. Since signal attenuation by the time span between the two π/2 pulses occurs by longitudinal (rather than transverse) relaxation and since this relaxation time, *T*_1_, may be much larger than *T*_2_, PFG NMR by the stimulated echo may attain notably larger observation times. 

In the PFG NMR measurements of this study we have applied the so-called 13-interval stimulated-echo pulse sequence [60]. By replacing the two fields gradient pulses of the basic experiment (Figure 1) by pairs of pulses with opposite field gradient amplitudes, separated by π pulses, in this technique a possibly disturbing influence due to internal field gradients can be further reduced.

The measurements have been performed with the home-built PFG NMR spectrometer FEGRIS 400 operating at a frequency of 400 MHz with a maximum field gradient intensity of 35 Tm^−^^1^ and with gradient widths δ varying from 0.3 ms to 0.6 ms [61,62]. The host-guest system under study was contained in glass samples of 7 mm diameter with a filling height of 10 mm. As guest molecules we used propane, at loadings corresponding to a concentration of 3 molecules per LTA super cage.

### 2.2. The Material under Study

**Figure 2:**
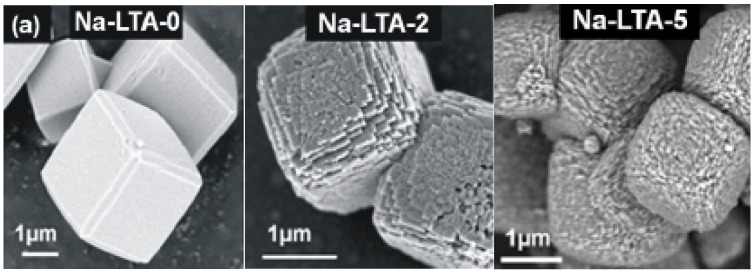
SEM images of crystals of (**a**) the purely microporous zeolite and (**b**) the mesoporous specimens. The images were taken from the external surface of calcined samples.

Following established procedures [63], the synthesis of mesoporous zeolite LTA was based on the application of 3-(Trimethoxysilyl)propylhexadecyldimethylammonium chloride (TPHAC) as an organosilane surfactant, giving rise to the formation of a mesopore network within the LTA microporous space. The TPHAC was obtained as a 56 wt% solution in methanol, following the synthesis procedure described in [64]. Three samples of LTA zeolite were hydrothermally synthesized at the gel compositions of 100 SiO_2_/333 Na_2_O/67.0 Al_2_O_3_/20000 H_2_O/n TPHAC, where n was varied from 0 to 2 and 5. The zeolite samples thus synthesized were denoted as Na-LTA-0, Na-LTA-2 and Na-LTA-5, respectively. Here, “Na” means the cationic form of LTA zeolite, and the numbers following “LTA” refer to the TPHAC mole numbers. Figure 2 shows typical SEM images of the thus produced zeolite crystallites. 

Ca^2+^ ion exchange was accomplished by dispersing 1 g of NaA-n in 200 mL 1M Ca(NO_3_)_2_∙4H_2_O solution for 3 h, with magnetic stirring at 333 K. This treatment was repeated three times in all, collecting sample by filtration each time. The ion-exchanged zeolite (now denoted as NaCa-LTA-0, 2 and 5) were dried in an oven at 373 K. 

Structure regularity was confirmed by X-ray diffraction as well as by field emission scanning electron microscopy (SEM, Figure 2) and transmission electron microscopy (TEM) [63]. From the micrographs, the mean crystal radius is found to be about 2 μm. Mesopore interconnectivity was confirmed by TEM of platinum nanowires [63] which were formed within the mesopore network by adopting a procedure introduced in reference [65]. The volume of the micro- and mesopore spaces and the mean diameter of the mesopores was determined from the N_2_ adsorption isotherms at 77 K, yielding a value of 0.2 mL g^−^^1^ for the micropore volume in all samples, and values of 0.15 mL g^−^^1^ and 0.35 mL g^−^^1^ for the mesoporous zeolites NaCa-LTA-2 and NaCa-LTA-5, respectively. The mean mesopore diameter results to be about 5 nm.

## 3. Results and Discussion

### 3.1. Measurements with the Purely Microporous Samples 

**Figure 3:**
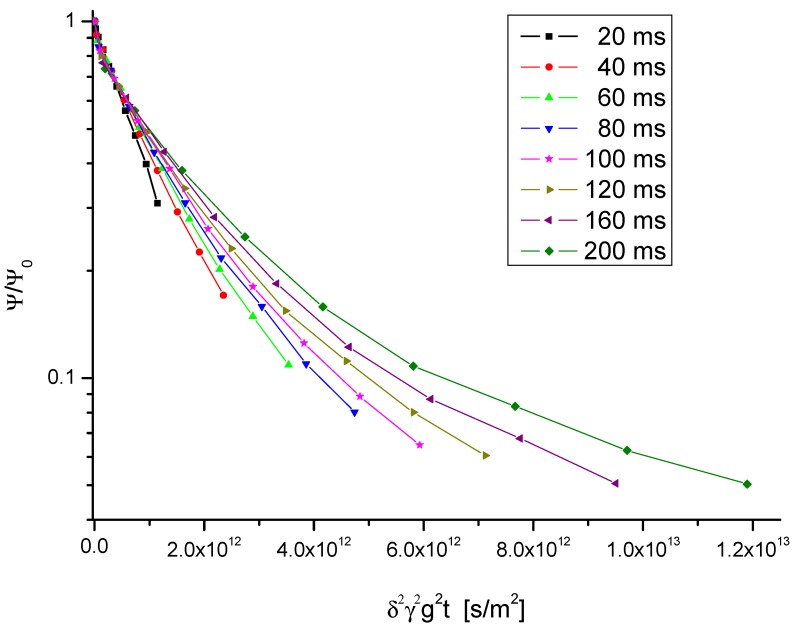
PFG NMR attenuation curves of propane in zeolite NaCa-LTA-0 at a temperature of 25 °C for different observation times.

Figure 3 shows typical examples of the primary data, namely the PFG NMR signal intensity plotted in a logarithmic representation as a function of the quantity *γ*^2^*δ*^2^*g*^2^*t*. We shall disregard, in our analysis, the flattening of the attenuation curves as appearing in the Figure 3 with large gradient intensities at attenuations down to 10%. These deviations from the behavior expected on the basis of Equation (8) can be referred to the contribution of a small amount of crystallites with smaller radii where, following Equation (7), the effective (restricted) diffusivities are shifted to smaller values. 

**Figure 4:**
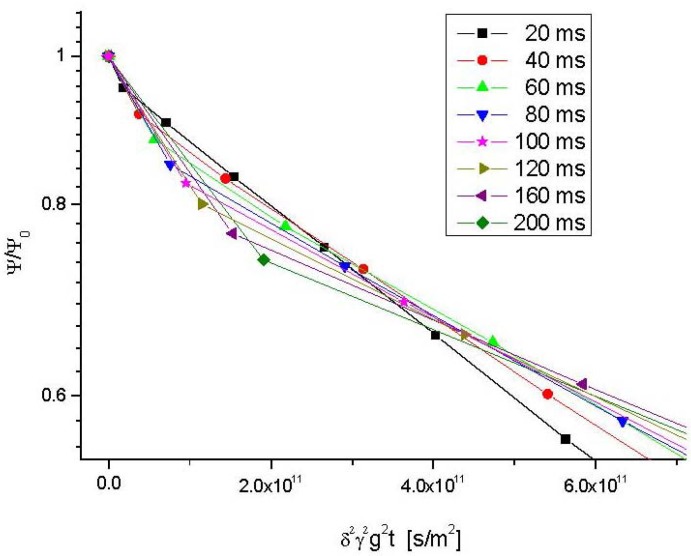
Scale-up of the beginning of the PFG NMR attenuation curves for propane in NaCa-LTA-0 at 25 °C as shown in Figure 3 for elaborating the distinction between the first fast and the second, more slowly decaying part of the attenuation curve. The second, more slowly decaying part of the attenuation curve originates from those molecules which, during time *t*, have remained in the interior of one crystallite.

**Figure 5:**
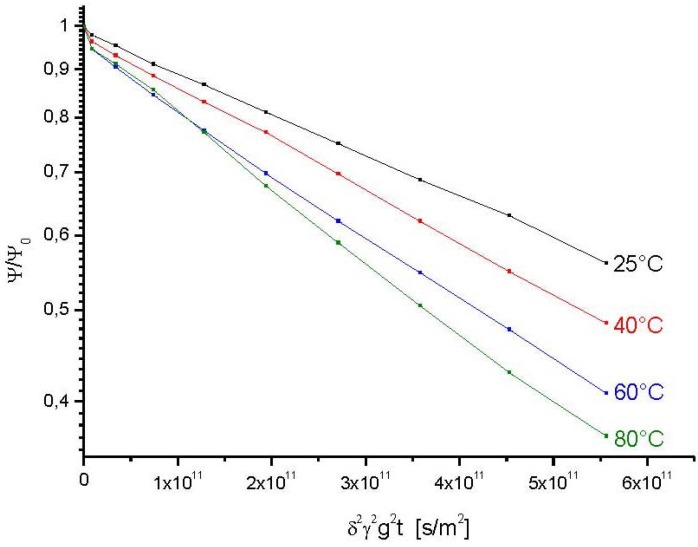
PFG NMR attenuation curves of propane in zeolite NaCa-LTA-0 for an observation time of 20 ms for different temperatures.

We rather concentrate on the first part of the attenuation curves. Examples are given in Figure 4 for different observation times at 25 °C and in Figure 5 for different temperatures at the smallest observation time (20 ms) considered with these samples. 

With Figure 4 and Figure 5, the PFG NMR attenuation curves may be correlated with the analytical expression given by Equation (8). In this way, from the relative contribution of the second, slowly decaying part one directly obtains the fraction *p*(*t*) of those molecules which, during *t*, have remained in the interior of one and the same crystallite. 

**Figure 6:**
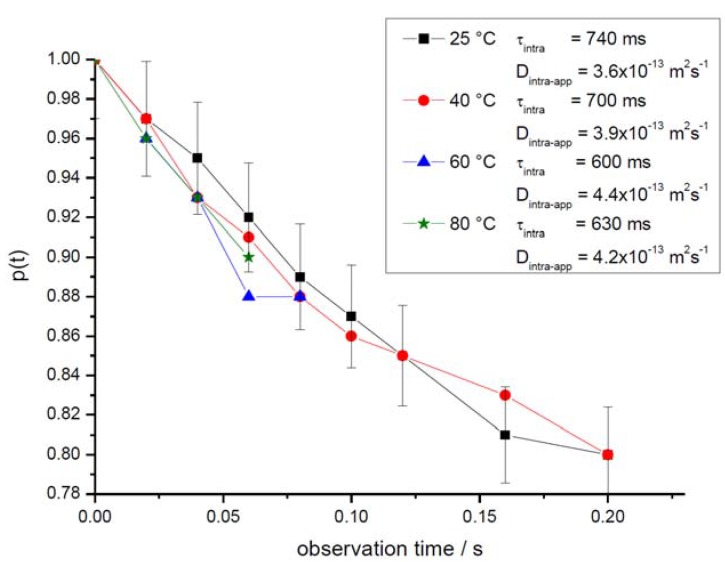
The fraction *p*(*t*) of molecules which, over the observation time *t,* remain in one and the same crystal (NMR tracer desorption (exchange) curve) as determined for different temperatures with propane as a guest molecule in the purely microporous zeolite NaCa-LTA-0. Indicated in the inset are the intracrystalline mean lifetimes following by applying Equations (10) and (12). Also shown are the (apparent) diffusivities which result via Equation (11) with the microscopically determined crystallite radius of about 2 μm if the tracer desorption curves were analyzed by implying diffusion-limited tracer exchange.

In complete agreement with our expectation, it can be seen from the representation of Figure (4) that the relative amount of molecules leaving the crystallites increases with increasing observation time. This leads, in turn, to decreasing values of *p*(*t*) (*i.e.*, the NMR tracer desorption (or “tracer exchange”) curves) as displayed in Figure 6.

It appears already from the representations with different temperatures in Figure 5 and becomes even more evident with the complete representation of the dependences of *p*(*t*) *vs.* *t* for different temperatures in Figure 6 that, as an unexpected and rather strange result, the rate of molecular exchange between different crystallites does not significantly vary with varying temperature. However, the accuracy of the underlying experimental data is rather limited as exemplified by the error bars given in Figure 6. It is, moreover, only a very small fraction of molecules (at the two higher temperatures of 60 °C and 80 °C not more than about 10%) which are considered. It is impossible, therefore, to deduce any meaningful conclusion out of these data. 

We are now going to consider the intracrystalline diffusivities as resulting from the slope of the second, slowly decaying part,

*D*_intra, eff._ = <*z*^2^(*t*)>_intra_/2*t* (13)

**Figure 7:**
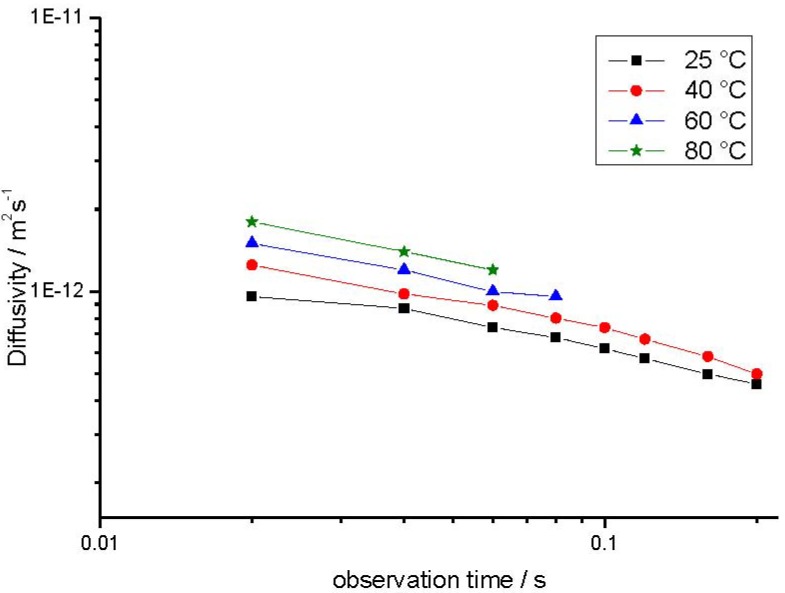
Effective intracrystalline diffusivity (Equation (13)) of propane in NaCa-LTA-0 as a function of the observation time *t* obtained at different temperatures.

<*z*^2^(*t*)>_intra_ denotes the mean value of the squares of the displacements in *z* direction within the crystals which for isotropic systems, like in the given case, is equal to <*r*^2^(*t*)>/3. Figure 7 provides an overview of the thus determined effective intracrystalline diffusivities.

Obviously, for sufficiently small times, *i.e.*, for <*r*^2^(*t*)> << *R*^2^, Equation (13) yields the true diffusivity since boundary effects due to encounters between the diffusing molecules and the crystal surface are negligible. Inversely, for sufficiently large times, the mean square displacement must be expected to be totally determined by the size of the crystals yielding *D*_restr._ = *R*^2^/5*t* (Equation (7)). In agreement with these considerations, the diffusivities are found to increase with decreasing observation time and one observes that, for sufficiently small observation times, diffusivities of the order of 10^−12^ m^2^s^−1^ are attained. These values nicely agree with the corresponding literature data [66] for self-diffusion of propane in NaCaA. 

The decrease of the effective intracrystalline diffusivity with increasing observation time can easily be attributed to the effect of confinement of mass transfer to the interior of the individual crystallites. As a consequence of this confinement, the effective intracrystalline diffusivities must be expected to approach a limiting value as provided by Equation (7). As a consequence of the inevitable distribution of the crystal sizes, this decrease becomes observable as soon as the mean diffusion paths get into the order of the radii of the smallest crystals. 

It is exactly this distribution in the crystal sizes which one has also to take into consideration on rationalizing the results shown in Figure 6. Note that all data refer to exchange phenomena with not more than 20% of the total amount of guest molecules. Again, this exchange will most likely occur within the smallest crystals so that, for the estimate of *D*_intra-eff_, presumably notably smaller radii than the microscopically determined 2 μm should be used. This would lead to even smaller values *D*_intra-eff_ and, hence, to an even larger discrepancy with the true intracrystalline (micropore) diffusivities as directly determined by PFG NMR with the smallest observation times. Intercrystalline molecular exchange is thus found to be notably affected by transport resistances on the surface of the individual crystallites. The occurrence of such resistances is well known from previous studies with comparable systems [67,68].

### 3.2. Measurements with the Mesoporous Samples 

**Figure 8:**
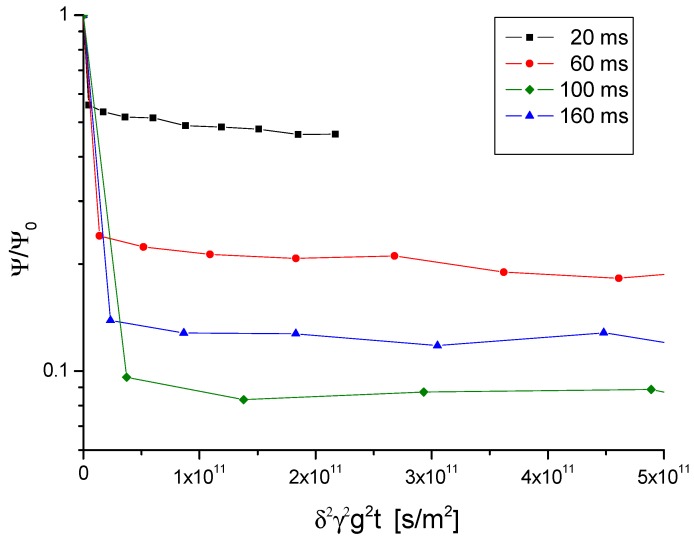
PFG NMR attenuation curves for propane in NaCa-LTA-2 for different observation times at 25 °C.

**Figure 9:**
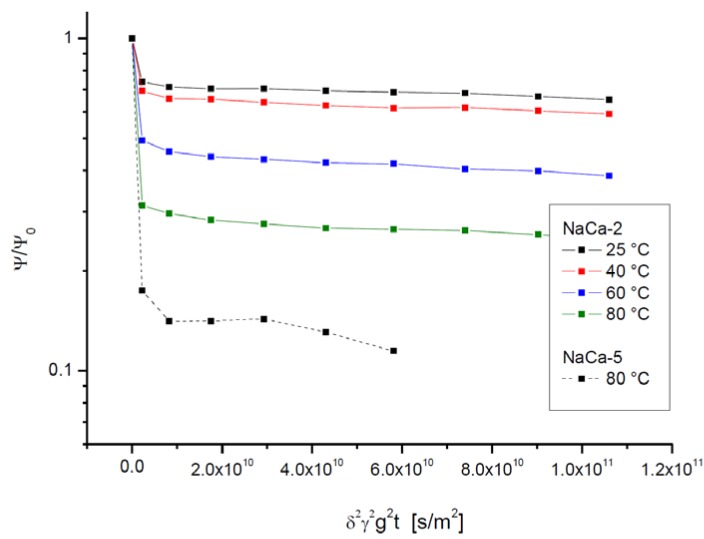
PFG NMR attenuation curves for propane in NaCa-LTA-2 and NaCa-LTA-5 at different temperatures for an observation time of 10 ms.

The PFG NMR attenuation plots for the mesoporous samples shown in Figure 8 and Figure 9 correspond to those plotted in Figure 4 and Figure 5 for the purely microporous ones. They most impressively demonstrate the dramatic acceleration of molecular exchange caused be the presence of the mesopores: While the first fast decay in the PFG NMR attenuation plots as an indication of molecular exchange is often only scarcely visible in the purely microporous zeolites, it dominates the PFG NMR attenuation curves in the mesoporous zeolites. In the sample with the larger mesopore content, NaCa-LTA-5, it is even at the shortest observation time impossible to accurately determine the relative fraction of molecules which have remained within one and the same crystallite. These samples had to be left, therefore, out of our considerations. 

The difference in mass transfer between the purely microporous and the mesoporous zeolites becomes particularly obvious in the NMR tracer desorption curves, *i.e.*, the plots of the time dependence of the relative amount *p*(*t*) of molecules which, at time *t*, are still in the same crystal in which they have been already at *t* = 0. While in the purely microporous samples (Figure 6) even for the longest observation time (200 ms) more than 80% of the guest molecules are found to still reside in one and the same crystal, in mesoporous NaCa-LTA-2 more than as much as 90% of the molecules (corresponding to a decrease of the relative amount *p*(*t*) of molecules, which have not yet left their crystallite, down to 0.1) are seen to have exchanged between different crystallites over already much smaller time intervals. In complete agreement with our expectation, molecular exchange with the surrounding is now clearly found to be notably accelerated with increasing temperature. 

**Figure 10:**
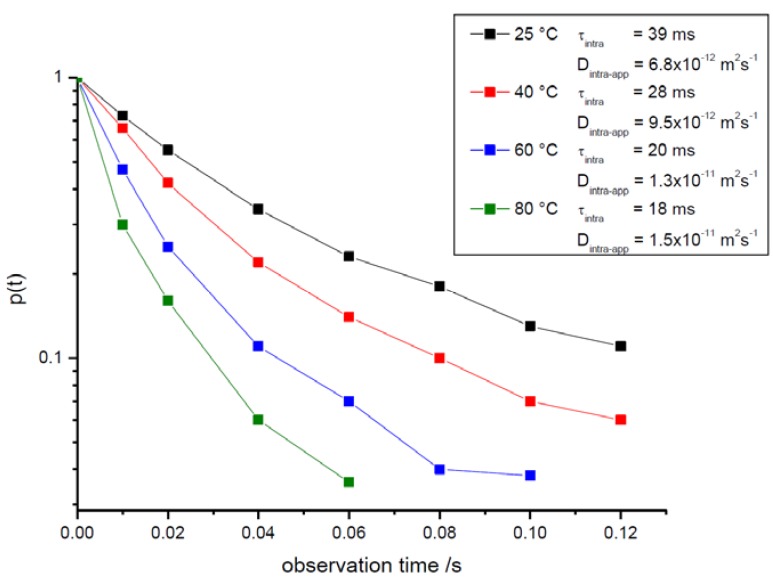
NMR tracer desorption curves for propane in mesoporous zeolite NaCa-LTA-2 for different temperatures. Indicated in the inset are the intracrystalline mean lifetimes following by applying Equations (10) and (12). Also shown are the (apparent) diffusivities which result via Equation (11) with the microscopically determined crystallite radius of about 2 μm if the tracer desorption curves were analyzed by implying diffusion-limited tracer exchange.

The inset of Figure 10 shows the intracrystalline mean life times (determined from the NMR tracer desorption curves via Equations (10) and (12)) and the apparent intracrystalline diffusivity (resulting from the NMR tracer desorption curves via Equation (11) for diffusion-limited tracer exchange). Transport acceleration by the mesopores is impressively documented by comparison with the data for the purely microporous specimen shown in Figure 6. Since mass transfer in the purely microporous specimen was found to be influenced by both intracrystalline diffusion and the permeation rate through surface barriers, transport enhancement in the mesoporous specimens is expected to be caused by an acceleration of both these processes. 

By adopting the effective medium approach [69,70], in references [18,71] mass transfer resistance on the external surface of nanoporous particles is demonstrated to be dramatically reduced by the formation of “holes” within an otherwise impenetrable surface layer. Exactly such “holes” in the surface are also created in the mesoporous materials so that the absence of any significant surface resistances may in fact be implied

**Figure 11:**
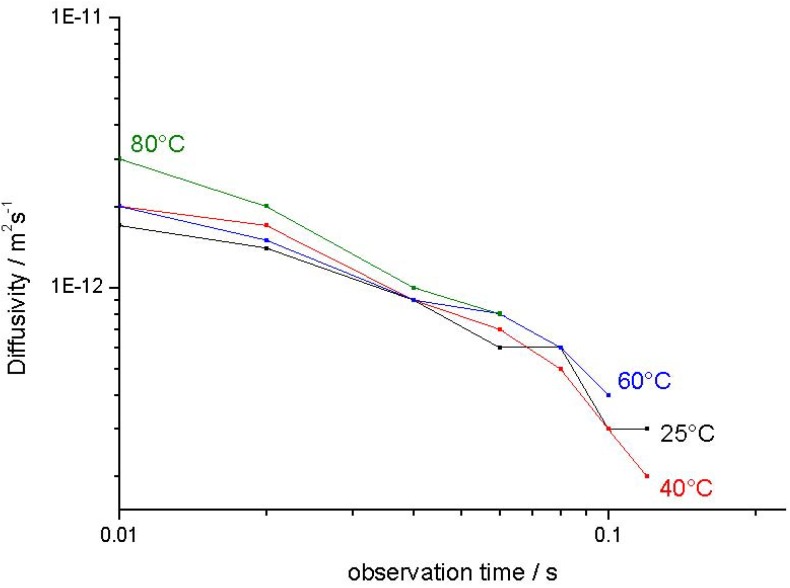
Order-of-magnitude estimate of the effective diffusivity of propane in NaCa-LTA-2 as resulting from the second slowly decaying part in the attenuation curves in Figure 8 and Figure 9.

Information about the intracrystalline diffusivity in the mesoporous material may, in principle, be deduced from the slope of the second, slowly decaying part of the attenuation curves. The values of *D*_intra,eff._ = <*z*^2^(*t*)>_intra_/2*t* thus resulting from the presentations in Figure 8 and Figure 9 are plotted in Figure 11 (corresponding to the representations of Figure 7 for the purely microporous materials). On assessing these values, however, one has to consider the limited accuracy of these measurements. The effective diffusivities had to be determined from the slope of the PFG NMR attenuation curves which, within the accessible measuring range, decay by not more than about 10%. This decay is already close to the relative error with which the data can be obtained so that the resulting data cannot be anything more than an order-of-magnitude estimate.

In addition, one must have in mind that, in general, the mesopore content will be different for different crystals. Since the second, flat part of the attenuation curve is due to the contribution of the “slow” molecules to the NMR signal, the effective intracrystalline diffusivities determined from this part must therefore be expected to be mainly associated with the diffusion in crystals with the smallest content of mesopores. Hence, it is not unexpected that transport enhancement by mesopores is not adequately reflected by the data shown in Figure 11.

Comparison of the experimental findings with the mesoporous zeolites NaCa-LTA-2 and NaCa-LTA-5 nicely illustrates the difficulties in correlating the textural properties of the mesoporous zeolites with the resulting diffusivities. With Figure 9 the relative amount p(t) of guest molecules at 25 °C which, after 10 s, are still accommodated by one and the same crystallite is seen to drop from about 0.75 for NaCa-LTA-2 to about 0.15 for NaCa-LTA-5. This corresponds, with Equations (11) and (12), to an increase of the intracrystalline diffusivity by a factor of about 7. Having in mind that the mesopore volume in NaCa-LTA-5 has increased by only slightly more than a factor of 2 and that the mean pore diameter remained essentially the same, a notably smaller effect should have been expected. On the basis of the present data, no definite explanation of this difference can be given. Most likely, however, the dramatic difference between NaCa-LTA-2 and NaCa-LTA-5 in the guest exchange rates must be taken as an indication of the existence of surface barriers in also the mesoporous zeolites and that, in NaCa-LTA-2, these resistances are still much more pronounced than in NaCa-LTA-5.

## 4. Conclusions

Owing to the dramatic difference in the respective NMR tracer desorption curves in Figure 6 and Figure 10, PFG NMR has been demonstrated to provide clear evidence for a most significant transport enhancement in mesoporous zeolites in comparison with their purely microporous counterpart. This evidence can be quantitated by both the intracrystalline mean life times (or, in other words, the intercrystalline exchange times) and the effective diffusivities deduced from the tracer desorption curves.

The evidence provided by this type of experiment was particularly impressive owing to the small size of the crystals used in these experiments. It was notably in the mesoporous specimens, that the thus attainable exchange rates were found to attain values which were easily accessible by the PFG NMR measurements. 

It was also, however, these enlarged exchange rates which excluded the option to reliably measure the intracrystalline diffusivities in the mesoporous zeolites. Measurements of this type require the application of larger crystals. Reference [72] reports about the first results of such studies.
